# Patients’ attitudes towards involvement of medical students in their care at university teaching hospitals of three public universities in Uganda: a cross sectional study

**DOI:** 10.1186/s12909-022-03576-4

**Published:** 2022-07-02

**Authors:** Amos Deogratius Mwaka, Seti Taremwa, Winnie Adoch, Jennifer Achan, Peruth Ainembabazi, Grace Walego, Moses Levi Ntayi, Felix Bongomin, Charles Benstons Ibingira

**Affiliations:** 1 Department of Medicine, School of Medicine, College of Health Sciences, Makerere University, Kampala, Uganda; 2 Department of Medicine, Faculty of Medicine, Gulu University, Gulu, Uganda; 3 School of Medicine, College of Health Sciences, Makerere University, Kampala, Uganda; 4 School of Public Health, College of Health Sciences, Makerere University, Kampala, Uganda; 5 Department of Medical Microbiology and Immunology, Faculty of Medicine, Gulu University, Gulu, Uganda; 6 Department of Anatomy, School of Biomedical Sciences, Makerere University, P.O Box 7072, Kampala, Uganda

**Keywords:** Medical students, Patients’ consent, Privacy, Clinical skills, Medical education

## Abstract

**Background:**

Comfort of patients with medical students is important and promotes appropriate clinical reasoning and skills development in the students. There is however limited data in this field in Uganda. In this study, we examined the attitudes and comfort of patients attending care at the medical and obstetrics/gynecology specialties in teaching hospitals of three public universities in Uganda.

**Methods:**

We conducted a cross sectional study among patients attending care at teaching hospitals for three public universities; Makerere University (Mak), Mbarara University of Science and Technology (MUST), and Gulu University (GU). Logistic regression was used to determine the magnitude of associations between independent and dependent variables. Two-sided p < 0.05 was considered statistically significant.

**Results:**

Eight hundred fifty-five patients participated in the study. Majority were aged 18 — 39 years (54%, *n* = 460), female (81%, *n* = 696) and married (67%, *n* = 567). Seventy percent (*n* = 599) of participants could recognize and differentiate medical students from qualified physicians, and had ever interacted with medical students (65%, *n* = 554) during earlier consultations. Regarding attitudes of patients towards presence of medical students during their consultations, most participants (96%; *n* = 818) considered involvement of medical students in patients’ care as essential ingredient of training of future doctors. Most participants prefer that medical students are trained in the tertiary public hospitals (80%; *n* = 683) where they attend care. Participants who were single/never married were 68% less likely to recognize and differentiate medical students (aOR = 0.32, 95%CI: 0.22 — 0.53) from other members of the healthcare team as compared with married participants. Participants with university education had 55% lower odds of being comfortable with presence of medical students during consultation compared to those with primary education (aOR = 0.45, 95%CI: 0.21 — 0.94). Participants from MUST teaching hospital had twofold higher odds of being comfortable with presence of medical students compared to participants from Mak teaching hospitals (aOR = 2.01; 95%CI: 1.20 — 3.39).

**Conclusion:**

Patients are generally comfortable with medical students’ involvement in their care; they prefer to seek care in hospitals where medical students are trained so that the students may contribute to their care. Medical students need to introduce themselves appropriately so that all patients can know them as doctors in training; this will promote patients’ autonomy and informed decisions.

**Supplementary Information:**

The online version contains supplementary material available at 10.1186/s12909-022-03576-4.

## Background

Direct contact with patients play a crucial role in the development of clinical reasoning, communication skills, and professional attitudes among medical students [1]. Student patient interactions at the bedside during teaching by the physicians remain the cornerstone for the proper training of future doctors and the practice of medicine. Sir William Osler taught doctors that “it is a safe rule to have no teaching without a patient for a text, and the best teaching is that taught by the patient himself” [2, 3]. Most patients often allow medical students to get involved in their care [4, 5]. In the United Kingdom, a study involving 278 patients attending general practice surgery consultation with and without a medical student present in six general practices in the Oxford area showed that majority of patients had positive attitudes towards involvement of medical students in their care, irrespective of the sex of the medical student. Only eight patients (3%) of all respondents demonstrated discomfort with presence of medical students during their consultations and procedures [4]. Similarly, majority of patients in a study at a genitourinary facility in Leeds, UK, that involved 250 male and 250 female patients were comfortable with the involvement of medical students in their care. Only 13% and 15% of women and men respectively expressed discomfort; most of the patients who declined were younger women and men, those visiting the clinic for the first time, and women with no children. They were uncomfortable with both male and female medical students [6]. Similar findings were reported from Australia where majority of patients were comfortable with involvement of medical students and recommended students be part and parcel of the medical team [7]. Other studies have showed that the level of global satisfaction of patients with their care did not vary between patients who consulted with their physicians alone and those who consulted in the presence of medical students [8, 9]. A study in the US among surgical patients showed that patients’ attitudes were favorable regarding participation of medical students in their care. The year of study or experience of the medical students did not significantly influence the decisions of the patients to allow medical students participate in their care. The patients reported that medical students answered their key questions and improved their satisfaction with care [10]. In the study by York et al., most patients would allow medical students participate in their future hospital care mainly because students provided most of the information they needed for their decisions making and were less in a hurry compared to the qualified healthcare professionals [10]. The finding that patients based quite a lot of their decisions on information got from students is important for the healthcare professionals during counseling and decision making especially if students provided inaccurate information upon which decisions are being made by the patients and their families. A study conducted at various departments in India involving 200 patients showed that majority (83.5%) of patients were comfortable with the presence of medical students among the hospital care team. Male patients were more positive and welcoming to the students compared to female patients. In regard to specialties, patients in the obstetrics and gynecology wards were more likely to reject student involvement in hospital procedures [11]. In a study in the United Arab Emirates, two hundred sixty-four women (87.1%) accepted student involvement; 158 women (59.8%) preferred female students. Comfort levels were significantly lower with male students in all skills that were tested particularly pelvic examination and the discussion of sexual problems [12]. Similar findings have been reported from Africa. In Tunisia, it was found that higher acceptance and comfort with medical students’ involvement in care was among male patients, patients aged more than 40 years, and those employed compared to women, patient aged under 40 and unemployed patients [13]. In Ethiopia, a study involving three hundred and ninety-two inpatients showed that participants were acceptable to involvement of medical students in their care. The level of acceptance varied with the specialty; medical (77.4%), surgical (72.0%) and gynecology ward (69.2%), although the differences were not statistically significant. Less than half (26.8%, *n* = 150) of patients expressed discomfort with the presence of medical students during their care [14].

A systematic review involving sixteen studies (1990 to 2010) showed that patients with emotional problems and those that needed an intimate examination were less likely to allow involvement of medical students in their care. The patients considered participation of medical students as important because it is the right thing to do as a contribution to the training of the students (altruism), but also because they get more time, a thorough physical examination, and receive better patient education as the students are being taught [15]. Patients who decline medical students’ involvement in their care express concerns with privacy especially during intimate examinations, inadequate students’ supervision, consultations involving emotional problems, and student level of training and therefore their perceived skillsets and competence [15, 16]. There are some evidence that women in the obstetrics and gynecology services have concerns with the skills of medical students in certain procedures including delivery and pelvic examinations. These concerns in some ways influence their decisions to accept involvement of medical students in their care [16]. Patients who negatively perceive involvement of medical students in their healthcare can provide false and inappropriate information simply for the fulfillment of the request of the medical students to clerk them [4]. False information from patients regarding symptoms and their evolution potentially misleads healthcare professionals including medical students in the evaluation and diagnosis of the patients. In a study of 222 women who accepted medical students and 78 who objected to medical students’ involvement in their care, 73% of those who accepted said they do so to support learning of the students, while 61% of those who objected raised concerns with their privacy as the main reasons to refuse medical students’ participation in their care [17].

In Uganda, medical students are trained in the regional, large private, and national referral hospitals that are meant for the provision of specialized healthcare to the populations [18, 19]. However, there is limited data from Uganda and sub–Saharan Africa generally on the attitudes and comfort of patients with the presence of medical students during their care. These data is important in informing training of medical students, and provision of quality acceptable medical services to patients. In this study we aimed to evaluate the perceptions, dispositions and willingness of patients attending gynecology, diabetes and hypertension clinics and wards regarding involvement of medical students in patients’ care in four selected public university teaching hospitals in Uganda.

## Methods

### Study setting

This study was conducted at the teaching hospitals for three public universities; Makerere University (Mak), Mbarara University of Science and Technology (MUST), and Gulu University (GU). Makerere University is the oldest of the three universities, followed by MUST then Gulu University. For Makerere University, we collected data from Kiruddu National Referral hospital for the medical specialty patients and Kawempe National Referral hospital for obstetrics – gynecology patients. Medical students in MUST are taught at Mbarara Regional Referral hospital where both medical and obstetrics – gynecology patients receive care. Medical students from Gulu University are taught from St. Mary’s hospital Lacor (Private Not for Profit) and Gulu Regional Referral hospital. We collected data from Gulu Regional Referral hospital where both medical and obstetrics—gynecology patients receive care. We did not collect data from the private not for profit hospital to avoid variation in characteristics of the patients due to ability to pay.

### Study design

This was a cross sectional study that used questionnaire for data collection.

### Study population, sample size and sampling procedure

The study population included patients with hypertension and diabetes attending the medical clinics and wards, and patients with gynaecology disorders attending care at the gynaecology clinics and wards of the selected university teaching hospitals. Prospective participants ought to be aged 18 years and above, of sound mind, and willing to provide written informed consents. Pregnant women were excluded from the study. Sample size was calculated based on the Kish Leslie formula for survey. The proportion of outcome of interest was estimated at 50% since we did not have similar studies from the region. The calculated sample size based on allowable error of 5% and a two sided level of significance at 5%, and alpha value of 1.96 was 384. We applied a design effect of 2 to account for inter-cluster variation which otherwise would lead to erroneous findings. Estimated sample size was therefore 384 × 2 = 768. An additional 12% (92) was considered for non-response and incomplete data. We therefore aimed to recruit 860 participants; 287 from each of the public university and half (430) from each of medical and obstetrics-gynaecology specialty. We used systematic random sampling to select participants for the study. The number of patients coming into the hospitals had reduced during the period of data collection because of travel restrictions related to the coronavirus 2019 (COVID-19) pandemic. We therefore sampled every third patient registered on the clinic days for the given hospital, and sampled every third patient among the new admissions. A list of registered patients was made as the patients reported to the clinics on the specific clinic days. The research assistants then selected every third patient on the list for inclusion into the study. The research assistants also visited the wards early morning (Monday to Friday) and made a list of all new patients. They similarly selected every third patient for inclusion into the study. The first participant was identified by simple random sampling of the first 3 patients. Thereafter, every 3rd patient on the list was approached for inclusion. If a selected patient declined, the next patient on the list was considered. Thereafter, the interval of every 3^rd^ patient was resumed. This process was conducted till the sample size was achieved for each teaching hospital.

### Data collection and management

We collected data during June through September 2020 when the Uganda National Council for Sciences and Technology (UNCST) allowed research involving patients to restart after a ban was laid in March 2020 because of the COVID−19 pandemic. We developed the tool for data collection based on experience and literature as well as adapting questions from questionnaires used in earlier studies [5, 20–22]. Questions were adjusted to make them relevant to the circumstances in Uganda. For example, questions regarding booking of appointments and keeping a given number of minutes for the appointment were removed as they do not apply to healthcare consultations in Uganda where patients walk in and meet any healthcare professionals assign to manage them without necessarily making a specific personal appointment. We piloted the tool with five patients from each of medical and obstetrics – gynaecology departments at the Makerere University teaching hospitals. We then reviewed the pilot data and adjusted the study tool accordingly before use in the main data collection. The 10 patients involved in the pilot phase were not included in the main study. The final English version of the tool (Additional file 1) was translated into Luo and Luganda to help the research assistants communicate the same thing to participants who did not understand English. No selected participants declined to participate.

Data collection was conducted by experienced graduate research assistants (RA), two of whom were pursuing master degrees in Public Health at Makerere University. The selected Research Assistants (RAs) were trained for two days on the study tool, objectives of the study, and consenting procedures; they were also provided a brief background on issues regarding patient doctor relationships and how the interactions can influence health−seeking including delay and advanced disease at diagnoses. These were to ensure quality data were collected. ST and FB supervised the research assistants during data collection. The RA collected data using android phones loaded with the Open Data Kit (ODK) software. Each RA interviewed patients independently. There were eight RAs: two (both female) in Gulu University, four (two female) in Makerere University, and two (one female) in Mbarara University of Science and Technology. Data from the ODK system of each RA was uploaded to an excel spreadsheet and reviewed by a biostatistician and GW. After data collection was completed, the biostatistician reviewed data from 12% of randomly selected participants to ensure data quality. There were no significant inconsistencies in the final dataset used for analyses.

### Data analysis

We conducted univariate analysis to describe the demographic characteristics of participants, and attitudes and dispositions of patients towards presence and involvement of medical students in their care. The results were reported as frequencies and percentages. Bivariate analysis using Chi square tests were conducted to determine associations between the binary outcome measures with socio-demographic correlates. Multivariate logistic regression models were applied on categorical variables with binary outcomes (Yes and No) to determine the magnitudes of associations between the binary outcome measures (e.g. questions 112 and 108) with selected independent variables. Statistical significance was set at two-sided p < 0.05. Effect measures reported were the odds ratios with their accompanying 95% confidence intervals. Question 112 in the questionnaire was used as the main outcome measure; “How do you feel about medical students being present while you are talking to the doctor about your problem?”, and the outcome was categorized as “Yes” and “No”. We also used question 113 as proxy and conducted sensitivity analyses using it. Question 113 was, “Would you allow medical student(s) to be present while you are talking to the doctor about your problem?” Question 108 was also used as an outcome measure regarding ability of participants to recognize medical students and differentiate them from qualified doctors. The question was; “When doctors come to see you for the problems that have brought you to the hospital, would you be able to tell whether some of the doctors are medical students?”.

## Results

### Characteristics of the participants

We enrolled a total of 855 study participants (99.4% response rate). Majority of participants were aged 18 – 39 years (53.8%; *n* = 460), female (81.4%; *n* = 696), and married (66.6%; *n* = 567). More than half (62.9%; *n* = 533) of participants had formal employment (Table 1).

**Table 1:** Socio-demographic characteristics of participants

Characteristics	Frequency	Percentage (%)
**Age (Years),** ***N*** **= 855**		
18–39 years	460	53.8
40–59 years	291	34.0
≥ 60	104	12.2
Missing	0	0
Median age: 38, IQR: 28 – 50 Mean age: 40.2, SD:14.7		
**Sex of participant,** ***N*** **= 855**		
Male	159	18.6
Female	696	81.4
**Marital status,** ***N*** **= 855**		
Married/Cohabiting	567	66.6
Single/Never married	93	10.9
Separated/Divorced	96	11.3
Widowed/Widower	95	11.2
Missing	4	
**Education Attainment,** ***N*** **= 842**		
Primary	327	38.8
Secondary	281	33.4
Tertiary	97	11.5
University	46	5.5
No formal education	91	10.8
Missing	13	
**Religion,** ***N*** **= 855**		
Catholic	328	38.5
Anglican	289	33.9
Islam	98	11.5
Pentecostal/Born again	128	16.0
Others	9	1.1
Missing	3	
**Employment,** ***N*** **= 848**		
No formal employment	315	37.2
Formal employment	533	62.8
Missing	7	
**University teaching hospital**		
Makerere University (Mak)	281	32.9
Mbarara University of Science and Technology (MUST)	286	33.5
Gulu University (GU)	288	33.7
**Specialty Clinic**		
Diabetic Clinic	382	44.7
Medicine Ward	58	6.8
Gynecology clinic	172	20.1
Gynecology ward	243	28.4

## Patients’ attitudes towards presence of medical students during consultations

Most participants (70.1%; *n* = 599) could recognize and differentiate medical students from qualified physicians, and more than half (64.8%, *n* = 554) had ever had medical students present during earlier consultations. Regarding attitudes of patients towards presence of medical students during their consultations, more than half (68.5%; *n* = 586) would not mind, while 13.8% (*n* = 118) would be eager/very eager with medical students during their consultations (Table 2).

**Table 2:** Attitudes towards presence of medical students during clinical encounters

Domains assessed	Frequency	Percentage (%)
**Participant can differentiate medical students from doctors**		
No	189	22.1
Yes	599	70.1
Not sure	63	7.4
Missing	4	
**Participant has ever had medical students present during consultation with their doctors**		
No	188	22.0
Yes	554	64.8
Not sure	113	13.2
**Feelings towards presence of medical students when patient is talking to the physician/doctor about his/her health problems**		
Extremely uneasy	67	7.8
Uneasy	84	9.8
Don’t mind	586	68.5
Eager	64	7.5
Very eager	54	6.3
**Allowable extent of participation of medical students in patient care**		
Medical history taking only	59	10.7
History taking and physical examinations	121	21.8
History taking, physical examinations and procedures	338	61.0
Observe physician/ doctor as she/he does her/his work of care	36	6.5
Will never allow medical students during my care		
**Circumstances under which participant would allow medical students to participate in his/her care**		
When she/he is requested by her nurse	74	13.4
When she/he is requested by her physician/doctor	223	40.3
When she/he is requested by her clinical/medical assistant	95	17.2
Will never allow medical students during my care	162	29.2
**Considerations made regarding involvement of medical students in patient care**		
**Patients’ cultural beliefs**		
No	786	91.9
Yes	69	8.1
**Patients’ religious beliefs**		
No	782	91.5
** Yes**	73	8.5
**Consultations take longer when medical students are present**		
No	598	70.0
Yes	257	30.1
**Patients’ personality**		
No	453	53.0
Yes	402	47.0
**When patients condition is not too serious/bad**		
No	383	44.8
Yes	472	55.2
**Prior experiences with medical students**		
No	554	64.8
Yes	301	35.2
**Sex of medical student**		
No	185	21.6
Yes	670	78.4
**Quality of care may be affected**		
No	595	69.6
Yes	260	30.4

The main considerations patients make in deciding whether or not medical students be involved in their care included perceived severity of illness (55.2%; *n* = 472) and sex (78.4%; *n* = 670) of the medical students. However, patients did not take into account prior experience with medical students (64.8%; *n* = 554), duration of consultation (70%; *n* = 598) and their own religious beliefs (91.5%; *n* = 782) (Table 2).

### Perceived importance of involving medical students in patients’ care

Most participants (95.7%; *n* = 818) considered involvement of medical students in patients’ care as an essential (very important and important) ingredient of training of future doctors. The majority of the participants would even prefer that medical students are trained in the public hospitals where they go for care (79.9%; *n* = 683) as opposed to the minority (23.7%; *n* = 203) who would prefer students to be trained in separate designated university teaching hospitals (Table 3).

**Table 3:** Perceived importance of involving medical students in patients’ care

Domain assessed	Frequency	Percentage
**How important is it for the training for future doctors, that medical students are present when patients are talking to their doctors/physicians about their problems?**		
Very important	682	79.8
Important	136	15.9
Not sure	27	3.2
Not important	8	0.9
Unnecessary	2	0.2
**How important is it for the training of future doctors that medical students examine patients**		
Very important	666	77.9
Important	149	17.4
Not sure	29	3.4
Not important	7	0.8
Unnecessary	4	0.5
**Is it preferable that medical students are trained in designated teaching hospitals separate from public hospitals**		
Most preferred	124	14.5
A lot preferred	79	9.2
Somewhat preferred	165	19.3
Not quite preferred	163	19.1
Not preferred at all	324	37.9
**Is it preferable that medical students are trained in the public hospitals including national and regional referral hospitals?**		
Most preferred	594	69.5
A lot preferred	89	10.4
Somewhat preferred	83	9.7
Not quite preferred	39	4.6
Not preferred at all	50	5.9

### Ability of patients to recognize medical students

Upon adjusting for age, sex, educational attainment and employment status, participants who were single/never married were 68% less likely to recognize and differentiate medical students (AOR = 0.32, 95%CI: 0.22 – 0.53) from other members of the healthcare team as compared with the married participants. On adjusting for the same socio-demographic factors above, participants from the newest of the three universities (Gulu University teaching hospital) were two and half times more likely to recognize and differentiate medical students from other members of the healthcare team (AOR = 2.51, 95%CI: 1.65 – 3.80) as compared to participants from Makerere University (Table 4).

**Table 4:** Factors associated with patients’ recognition of medical students

	**Recognizes medical students**		**Crude Odds ratio**	**Adjusted Odds ratio (95%CI)**^a^
**Characteristics**	**Yes** ***N*** **(%)**	**No** ***N*** **(%)**		
**Age of participants (Years)**				
18 – 39	310 (51.8)	147 (58.3)	1.00	1.00
40 – 59	218 (36.4)	72 (28.6)	1.44 (1.03—2.00)	1.19 (0.80—1.76)
≥ 60	71 (11.9)	33 (13.1)	1.02 (0.65—1.61)	1.01 (0.57—1.8)
**Sex of participant**				
Male	121 (20.2)	38 (15.1)	1.00	1.00
Female	478 (79.8)	214 (84.9)	0.70 (0.47—1.05)	1.04(0.64—1.71)
**Marital status**				
Married/cohabiting	416 (69.7)	147 (58.8)	1.00	1.00
Single/never married	47 (7.9)	46 (18.4)	**0.36 (0.23—0.56)**	**0.32 (0.2—0.53)**
Separated/divorced	63 (10.6)	33 (13.2)	0.67 (0.42—1.07)	0.86 (0.53—1.40)
Widowed/widower	71 (11.9)	24 (9.6)	1.05 (0.63—1.72)	1.71 (0.93—3.15)
**Education attainment**				
Primary education	215 (36.5)	111 (44.4)	1.00	1.00
No formal education	53 (9.0)	37 (14.8)	0.74 (0.46- 1.19)	0.67 (0.40—1.13)
Secondary education	206 (35.0)	74 (29.6)	**1.44 (1.01—2.04)**	**1.87 (1.28—2.74)**
Tertiary education	76 (12.9)	21 (8.4)	**1.86 (1.09—3.20)**	**2.32 (1.30—4.12)**
University	39 (6.6)	7 (2.8)	**2.88 (1.24—6.69)**	**4.15 (1.68—10.23)**
**Employment status**				
No formal employment	191 (32.1)	123 (49.2)	1.00	1.00
Formal employment	405 (68.0)	127 (50.8)	**2.05 (1.51—2.79)**	**1.79 (1.30—2.49)**
**University hospital**				
Makerere University	164 (27.4)	114 (45.2)	1.00	1.00
Mbarara University (MUST)	203 (33.9)	83 (32.9)	**1.70 (1.19—2.41)**	1.30 (0.88—1.93)
Gulu University	232 (38.7)	55 (21.8)	**2.93 (1.98—4.33)**	**2.51 (1.67—3.80)**
**Specialty**				
Gynecology	274 (45.7)	138 (54.8)	1.00	1.00
Medical	325 (54.3)	114 (45.2)	**1.44 (1.07—1.93)**	1.36 (0.900—2.05)

*MUST*  Mbarara University of Science and Technology

^a^Adjusted for all factors on table

Bold  Factors statistically significant

### Factors associated with patients’ dispositions towards presence of medical students

The majority of participants (82.3%; *n* = 704) were comfortable with medical students’ presence during their consultations. After adjusting for age, sex, marital status, and employment, participants with university education had 55% less odds of being comfortable with presence of medical students during consultation compared to those with primary education (adjusted odds ratio (aOR = 0.45, 95%CI: 0.21 — 0.94). On adjusting for age, sex, marital status, educational and employment status, participants from MUST teaching hospital had twofold higher odds of being comfortable with presence of medical students compared to participants from Makerere university teaching hospitals (aOR = 2.01; 95%CI: 1.20 — 3.39). However, age, sex, marital status and employment status were not significantly associated with patients’ comfort with the presence of medical students during clinical consultations (Table 5).

**Table 5:** Factors associated with comfort of patients with presence of medical students during consultations

	**Feels comfortable**		**Crude Odds ratio (95%CI)**	**Adjusted Odds ratio (95%CI)**^a^
**Characteristics**	**Yes,** ***N*** **(%)**	**No,** ***N*** **(%)**		
**Age of participants (Years)**				
18 – 39	364 (51.7)	96 (63.6)	1.00	1.00
40 – 59	249 (35.4)	42 (27.8)	**1.56 (1.05—2.33)**	1.46 (0.92—2.30)
≥ 60	91 (12.9)	13 (8.6)	1.85 (0.99- 3.45)	1.89 (0.91—3.95)
**Sex of participant**				
Male	133 (18.9)	26 (17.2)	1.00	1.00
Female	571 (81.1)	125 (82.8)	0.89 (0.56—1.42)	1.08 (0.64—1.80)
**Marital status**				
Married/cohabiting	463 (66.1)	104 (68.9)	1.00	1.00
Single/never married	74 (10.6)	19 (12.6)	0.87 (0.51—1.51)	1.09 (0.61—1.96)
Separated/divorced	81 (11.6)	15 (9.9)	1.21 (0.67—2.19)	1.15 (0.63—2.1)
Widowed/widower	82 (11.7)	13 (8.6)	1.42 (0.76—2.64)	1.05 (0.51—2.16)
**Education attainment**				
Primary education	280 (40.4)	47 (31.7)	1.00	1.00
Secondary education	221 (31.9)	60 (40.3)	**0.62 (0.41—0.94)**	0.69 (0.45—1.07)
Tertiary education	84 (12.1)	13 (8.7)	1.08 (0.56—2.10)	1.11 (0.57 -2.16)
University	33 (4.8)	13 (8.7)	0.43 (0.42—1.47)	**0.45 (0.21- 0.94)**
No formal education	75 (10.8)	16 (10.7)	0.789 (0.42—1.47)	0.69 (0.36—1.32)
**Employment status**				
No formal employment	257 (36.9)	58 (38.4)	1.00	1.00
Formal employment	440 (62.85)	93 (61.6)	1.07 (0.74—1.53)	1.08 (0.74—1.58)
**University hospital**				
Makerere University	229 (32.5)	52 (34.4)	1.00	1.00
Mbarara University (MUST)	256 (36.4)	30 (19.9)	**1.94(1.19–3.15)**	**2.01(1.20—3.39)**
Gulu University	219 (31.1)	69 (45.7)	0.72(0.48–1.08)	0.71 (0.46 -1.09)
**Specialty**				
Gynecology	334 (47.4)	81 (53.6)	1.00	1.00
Medical	370 (52.6)	70 (46.4)	1.28 (0.900—1.82)	1.04 (0.65—1.67)

*MUST*  Mbarara University of Science and Technology

^a^Adjusted for all factors on table

Bold  Factors statistically significant

### Influence of specialty on patient’s comfort with the presence of medical students during consultation

On adjusting for age, sex, marital status, education and employment status, patients from the medical departments of MUST had nearly threefold higher odds of being comfortable with the presence of medical students during consultations (aOR = 2.83, 95%CI: 1.24 – 6.49) compared to those from Makerere university teaching hospitals. However, no socio-demographic factors were associated with patients’ comfort with the presence of medical students among the gynecology patients in both MUST and GU teaching hospitals as compared to patients from Mak teaching hospital (Table 6 and 7).

**Table 6:** Factors associated with comfort of patients in the medical specialty with the presence of medical students during consultations

	**Feels comfortable**		**Crude Odds ratio (95%CI)**	**Adjusted Odds ratio (95%CI)**^a^
**Characteristics**	**Yes** ***N*** **(%)**	**No** ***N*** **(%)**		
**Age of participants (Years)**				
18—39	89 (24.1)	25 (53.7)	1.00	1.00
40—59	199 (53.8)	34 (48.6)	1.64 (0.93—2.92)	1.30 (0.68—2.50)
≥ 60	82 (22.2)	11 (15.7)	2.09 (0.97—5.52)	1.86 (0.77—4.48)
**Sex of participant**				
Male	133 (36.0)	26 (37.1)	1.00	1.00
Female	237 (64.1)	44 (62.9)	1.05 (0.62—1.79)	1.05 (0.59—1.89)
**Marital status**				
Married/cohabiting	238 (64.3)	47 (67.1)	1.00	1.00
Single/never married	27 (7.3)	8 (11.4)	0.67 (0.29—1.56)	0.92 (0.36—2.39)
Separated/divorced	39 (10.5)	8 (11.4)	0.96 (0.42—2.19)	0.86 (0.36—2.06)
Widowed/widower	66 (17.8)	7 (10.0)	1.86 (0.80—4.31)	1.7 (0.67—4.30)
**Education attainment**				
Primary education	152 (41.6)	22 (31.8)	1.00	1.00
Secondary education	101 (27.7)	22 (31.9)	0.66 (0.35—1.26)	0.79 (0.4—1.58)
Tertiary education	46 (12.6)	8 (11.6)	0.83 (0.35—1.99)	0.90 (0.36—2.22)
University	18 (4.9)	7 (10.1)	**0.37 (0.14—0.99)**	0.38 (0.133—1.07)
No formal education	48 (13.2)	10 (14.5)	0.69 (0.31—1.57)	0.62 (0.26—1.46)
**Employment status**				
No formal employment	116(31.7)	28(40.0)	1.00	1.00
Formal employment	250(68.3)	42(60.0)	**1.43 (0.85–2.43)**	**1.62 (0.92–2.87)**
**University hospital**				
Makerere University	114 (30.8)	25 (35.7)	1.00	1.00
Mbarara University (MUST)	137 (37.0)	11 (15.7)	**2.73 (1.29—5.79)**	**2.83 (1.24—6.49)**
Gulu University	119 (32.2)	34 (48.6)	0.77 (0.43—1.37)	0.80 (0.42—1.51)

*MUST*  Mbarara University of Science and Technology

^a^Adjusted for all factors on table

Bold  Factors statistically significant

**Table 7:** Factors associated with comfort of patients in the gynecology specialty with the presence of medical students during consultations

	**Feels comfortable**		**Crude Odds ratio (95%CI)**	**Adjusted Odds ratio (95%CI)**^a^
**Characteristics**	**Yes** ***N*** **(%)**	**No** ***N*** **(%)**		
**Age of participants (Years)**				
18 – 39	275 (82.3)	71 (87.7)	1.00	1.00
40 – 59	50 (15.0)	8 (9.9)	1.61 (0.73—3.56)	1.73 (0.69—4.35)
≥ 60	9 (2.7)	2 (2.5)	1.16(0.25–5.5)	2.51 (0.35—17.87)
**Sex of participant**				
Female	334	81	-	-
Male	-	-	-	-
**Marital status**				
Married/cohabiting	225 (68.2)	57 (70.4)	1.00	1.00
Single/never married	47 (14.2)	11 (13.6)	1.08 (0.53—2.22)	1.11 (0.51—2.46)
Separated/divorced	42 (12.7)	7 (8.6)	1.52 (065—3.56)	1.31 (0.44 -3.15)
Widowed/widower	16 (4.9)	6 (7.4)	0.68 (0.25—1.80)	0.30 (0.78—1.18)
**Education attainment**				
Primary education	128 (39.0)	25 (31.3)	1.00	1.00
Secondary education	120 (36.6)	38 (47.5)	0.62 (0.35—1.08)	0.6 (0.33—1.07)
Tertiary education	38 (11.6)	5 (6.3)	1.48 (0.53—4.14)	1.44 (0.500—4.14)
University	15 (4.6)	6 (7.5)	0.49 (0.17—1.38)	0.46 (0.15—1.40)
No formal education	27 (8.1)	6 (7.5)	0.88 (0.33—2.35)	0.87 (0.29—2.61)
**Employment status**				
No formal employment	141 (42.6)	30 (37.0)	1.00	1.00
Formal employment	190 (57.4)	51 (63.0)	0.79 (0.48—1.31)	0.81 (0.48—1.38)
**University hospital**				
Makerere University	115 (34.4)	27 (33.3)	1.00	1.00
Mbarara University (MUST)	119 (35.6)	19 (23.5)	1.47 (0.78—2.79)	1.64 (0.81—3.3)
Gulu University	100 (30.0)	35 (43.2)	0.67 (0.38—1.19)	0.68 (0.37—1.26)

*MUST* Mbarara University of Science and Technology

^a^Adjusted for all factors on table

### Patients’ preferred hospitals for training of medical students

Age, sex, marital status, educational attainment and employment status of participants were not significantly associated with their preference as to where medical students should be trained in separate designated university teaching hospitals or in the tertiary public hospitals as it is the case currently. Upon adjusting for the socio-demographic factors, participants from MUST had 63% less odds of preferring medial students to be trained in designated and separate university teaching hospitals (AOR = 0.27, 95%CI: 0.17 — 0.44) as compared to the participants from Makerere University (Table 8). However, when asked as to whether medical students be trained in the tertiary public hospitals as opposed to separate university teaching hospitals, most participants (79.9%; *n* = 683) answered to the affirmative. On adjusting for the socio-demographic factors, participants from both GU and MUST teaching hospitals had 2 – 8 folds higher odds (AOR = 2.27 – 8.14, 95%CI: 1.50 – 14.50) compared to those from Mak teaching hospitals to prefer training of medical students to be done in the tertiary public hospitals (Table 9).

**Table 8:** Factors associated with patients’ disposition as to whether medical students be trained in the tertiary public hospitals

	**University teaching hospitals to be autonomous**		**Crude Odds ratio (95%CI)**	**Adjusted Odds ratio (95%CI)**^a^
**Characteristics**	**Yes** ***N*** **(%)**	**No** ***N*** **(%)**		
**Age of participants (Years)**				
18 – 39	113 (55.7)	347 (53.2)	1.00	1.00
40 – 59	64 (31.5)	227 (34.8)	0.87 (0.61—1.22)	0.82 (0.54—1.23)
≥ 60	26 (12.8)	78 (12.0)	1.02 (0.63—1.67)	0.99 (0.5 5- 1.81)
**Sex of participant**				
Male	42 (20.7)	117 (17.9)	1.00	1.00
Female	161 (79.3)	535 (82.1)	0.84 (0.57—1.24)	0.85 (0.54—1.23)
**Marital status**				
Married/cohabiting	140 (69.3)	427 (65.8)	1.00	1.00
Single/never married	23 (11.39)	70 (10.8)	1.00 (0.60—1.67)	0.85 (0.49- 1.47)
Separated/divorced	18 (8.9)	78 (12.0)	0.70 (0.41—1.22)	0.77 (0.44—1.34)
Widowed/widower	21 (10.4)	74 (11.4)	0.86 (0.51—1.46)	1.05 (0.57—1.93)
**Education attainment**				
Primary education	76 (38.2)	251 (39.0)	1.00	1.00
Secondary education	67 (33.7)	214 (33.3)	1.03 (0.71—1.51)	0.99 (0.67—1.46)
Tertiary education	24 (12.1)	73 (11.4)	1.09 (0.64—1.84)	1.10 (0.64—1.89)
University	16 (8.0)	30 (4.7)	1.76 (0.91—3.42)	1.75 (0.88—3.49)
No formal education	16 (10.8)	75 (11.7)	0.70 (0.39—1.28)	0.7 (0.64—1.89)
**Employment status**				
No formal employment	78 (38.8)	237 (36.6)	1.00	1.00
Formal employment	123 (61.2)	410 (63.4)	0.91 (0.66–1.26)	0.88 (0.62—1.24)
**University hospital**				
Makerere University	83 (40.9)	198 (30.37)	1.00	1.00
Mbarara University (MUST)	33 (16.3)	253 (38.8)	**0.31 (0.19—0.49)**	**0.27 (0.17—0.44)**
Gulu University	87 (42.9)	201 (30.8)	1.03 (0.72—1.48)	0.99 (0.67—1.46)
**Specialty**				
Gynecology	96 (47.3)	319 (48.9)	1.00	1.00
Medical	107 (52.7)	333 (51.1)	1.07 (0.78—1.47)	1.15 (0.74—1.76)

*MUST* Mbarara University of Science and Technology

^a^Adjusted for all factors on table

Bold Factors statistically significant

**Table 9:** Medical students to be trained in the regional and national referral hospitals

	**Regional and national referral hospitals to be used for training**		**Crude Odds ratio (95%CI)**	**Adjusted Odds ratio (95%CI)**^a^
**Characteristics**	**Yes preferred** ***N*** **(%)**	**Not preferred** ***N*** **(%)**		
**Age of participants (Years)**				
18 – 39	374 (54.8)	86 (50.0)	1.00	1.00
40 – 59	227 (33.2)	64 (37.2)	0.82 (0.57—1.17)	0.81 (0.53—1.25)
≥ 60	82 (12.0)	22(12.8)	0.86 (0.51—1.45)	1.03 (0.54—1.96)
**Sex of participant**				
Male	128 (18.7)	31 (18.0)	1.00	1.00
Female	555 (81.3)	141 (82.0)	0.95 (0.62—1.47)	1.10 (0.67—1.80)
**Marital status**				
Married/cohabiting	461 (67.7)	106 (62.4)	1.00	1.00
Single/never married	75 (11.0)	18 (10.6)	0.96 (0.55—1.67)	1.02 (0.56—1.86)
Separated/divorced	75 (11.0)	21 (12.4)	0.82 (0.48—1.39)	0.77 (0.45—1.33)
Widowed/widower	70 (10.3)	25 (14.7)	0.64 (0.39—1.07)	0.63 (0.34—1.14)
**Education attainment**				
Primary education	265 (39.4)	62 (36.5)	1.00	1.00
Secondary education	220 (32.7)	61 (35.9)	0.84 (0.57—1.25)	0.81 (0.53—1.23)
Tertiary education	78 (11.6)	19 (11.2)	0.96 (0.54—1.70)	0.93 (0.51—1.7)
University	39 (5.8)	7 (4.1)	1.30 (0.56—3.06)	1.06 (0.44—2.56)
No formal education	70 (10.4)	21 (12.4)	0.78 (0.44—1.37)	0.99 (0.54—1.81)
**Employment status**				
No formal employment	235 (34.6)	80 (47.6)	1.00	1.00
Formal employment	445 (65.4)	88 (52.4)	**1.72 (1.22—2.43)**	**1.69 (1.18—2.42)**
**University hospital**				
Makerere University	184 (26.9)	97 (56.4)	1.00	1.00
Mbarara University (MUST)	268 (39.2)	18 (10.5)	**7.85 (4.42—13.93)**	**8.14 (4.5 7—14.50)**
Gulu University	231 (33.8)	57 (33.1)	**2.14 (1.45—3.14)**	**2.27 (1.5—3.44)**
**Specialty**				
Gynecology	328 (48.0)	87 (50.6)	1.00	1.00
Medical	355 (52.0)	85 (49.4)	1.11 (0.79—1.55)	1.38 (0.87—2.19)

*MUST *Mbarara University of Science and Technology

^a^Adjusted for all factors on table

Bold Factors statistically significant

## Discussions

We present insights from sub Saharan Africa regarding patients’ attitudes towards and comfort with medical students during clinical consultations and care. In our knowledge, this is the first study from Uganda since the inception of the first medical school at Makerere University in 1923 that has assessed patients’ comfort with the involvement of medical students in their care in three university teaching hospitals. We found that most patients could recognize and differentiate medical students from doctors. Majority of the participants had had previous experiences with medical students, and do not mind the involvement of medical students in their care nor are they bothered with the increased consultation time because the physicians are teaching medical students during consultations. Participants prefer to attend care in the university teaching hospitals where medical students are trained than in equivalent level hospitals where students are not. The sex of the medical student was not an important consideration in deciding whether or not a medical student should be involved in a patient’s care. The patients’ level of comfort with medical students did not significantly vary between patients attending care at the medical and obstetrics-gynecology departments. Highly educated patients from the medical department were less likely to be comfortable with the involvement of medical students in their care. The few participants who were uncomfortable with involvement of medical students in patients’ care were concern with invasion of privacy by the unqualified medical students. The patients from Makerere University teaching hospitals situated in the capital city were significantly more likely to be uncomfortable with involvement of medical students in patients’ care.

In this study, majority of participants were young (aged less than 40 years), female, married and with some formal employments. The age distribution of participants in this study is similar to other studies in this field of research. For example, a study in Canada that involved 625 patients from various specialties had a mean age of 39 years, with the majority aged 30 – 65 years. Majority of the patients (62%) in that study were female [23]. In the US, a study in the Midwest involving 213 obstetric gynecology patients had a mean age of 34.9 years [20]. In this study, age of participants was not statistically associated with being comfortable with involvement of medical students in patient care. While it could be expected that older patients would be uncomfortable with involvement of medical students (most are young) in their care, our data do not show that. Earlier studies showed that both outpatients and older hospitalized patients have positive attitudes towards medical students’ involvement in their care [24]. Although more context specific data are needed, our finding that age of patients does not determine patients’ comfort and acceptance of medical students’ involvement in patient care means that the deployment of medical students shall therefore not be restricted by the age of the patients.

Most participants in this study were able to recognize and differentiate medical students from the qualified medical doctors. Majority of them had ever had medical students present in their previous consultations and healthcare. The participants who were married, with higher educational attainment and those formally employed were more likely to recognize medical students. Our finding coheres with most studies in which majority of patients recognize medical students. However, results from a few studies show that a varying proportion of patients don’t know how to differentiate qualified healthcare professionals from medical students [10, 13]. For example, in Tunisia, up to 78% of patients did not realize that medical students were involved in their care [13]. In Australia, a study among women attending antenatal care reported that more than half of the women (54%; *N* = 625) had challenges differentiating medical students from other health professionals cadres [25]. It is important that patients are told that medical students are involved in their care. The patients should consciously consent to involvement of medical students in their care. They should as well be told the roles of medical students in their care.

The majority of participants were comfortable with involvement of medical students in their care. They did not feel that presence of medical students would adversely affect the quality of care, nor were they concern with the longer duration of consultation when medical students are present. This positive finding regarding acceptance of medical students by patients in university teaching hospitals is quite encouraging. Our finding is similar to results from other studies from both the high-income countries (HIC) and low- and -middle income countries (LMIC) where patients across specialties have shown acceptance for medical and other healthcare students to be involved in patients’ care as part of the students’ training. Data from the high-income countries show that medical students are highly accepted (55 to 95% acceptance) in accident and emergency services but not as much among pregnant women, especially during intrapartum care [26–29]. Similarly, data from the LMIC also show high level of acceptance of medical students’ involvement in patients’ care. For example, in Ethiopia, 69.2% – 77.4% accepted medical students to participate in their care [13, 14]. High level of acceptance have been also reported from the Middle East; for example, in a study involving patients from various specialties in Saudi Arabia, patients were generally acceptable to students’ involvement in their care. Refusal rate was only 11% – 43%, mainly in the obstetrics—gynecology specialty [30]. Acceptance of medical students is higher for non-invasive contacts including reading patients’ files, observing doctors during ward rounds, and taking history than with intimate procedures including digital rectal exams, vaginal deliveries and episiotomy repairs, and pelvic examinations [13, 30]. Patients accept medical students to participate in their care because they want to contribute to the learning of the students and making of future doctors, companionship, and because they learn quite significantly about their own health states from the medical students who often give significant time to patients. Patients also feel that they learn more when the doctors are teaching medical students during consultations [16, 20, 23, 25, 28, 31]. Majority of patients concur that patients-medical students’ interactions is a critical factor in training of competent future doctors [23]. Participation of medical students in patients’ care was considered a worthwhile learning experience for the students [29]. Similar findings were reported from Australia, where 96% of patients (*N* = 248) acknowledged the importance of students’ involvement in patients’ care as part of their training [7]. Patients’ contact under different clinic settings is an invaluable and inseparable component of appropriate medical training to groom competent medical doctors and other healthcare professionals’ personnel. The patient-medical student interactions during training provides a firm irreplaceable platform for the development of clinical skills, patient-physicians communications, and ethical skills necessary for their practices in the future.

We found that participants with higher education standards were less comfortable with involvement of medical students in their care. However, demographic characteristics of participants including age, sex, marital and employment status were not significantly associated with attitudes and comfort with involvement of medical students in patients’ care. Our findings are different from that of a study from Tunisia, where it was found that higher acceptance and comfort with medical students’ involvement in care was among male patients, patients aged more than 40 years, and those employed compared to women, patient aged under 40 and unemployed patients [13]. Our findings also differs from that of Hartz et al. [20] which showed that patients’ education level did not influenced their decisions to allow involvement of medical students in their care in general. However, they reported that level of education significantly influenced acceptance and comfort level with medical students during intimate examinations including pelvic examinations and performance of Pap smear among women. Women with higher education achievement are more willing to accept medical students’ involvement than the less educated women [20]. Our findings also differ from results of a study in Australia, which showed that obstetrics and gynecology patients (*n* = 255) aged less than 40 years, and those who were inpatients were significantly more likely to be satisfied with involvement of medical students in their care. Satisfaction was higher among patients seen by female medical students (86%) compared with male students (74%) [7]. Majority of medical students maybe young. In our study, majority of our patients were also young, with median age 38 years (54% younger than 40 years). This could explain why age did not feature as a significant factor in determining patients’ comfort with medical students’ involvement. There was no significant difference by specialty regarding the level of acceptance and comfort with medical students except for education attainment. Passaperuma et al. [23] also found no inter-specialty differences regarding patients’ comfort and acceptance of medical students’ involvement in their care. However a large study involving 932 participants from 14 teaching hospitals in Kuwait showed significant inter-specialty difference in acceptance of medical students’ involvement in patients’ care. While acceptance was highest in the pediatrics specialty, refusal was highest in the obstetrics gynecology specialty [32]. We recommend more studies in sub Saharan Africa across various specialties to shine more light on the effects of subspecialties and patients’ comfort with medical studies.

In this study, majority of participants said they would put into considerations the sex of the medical student (78%) and the seriousness of the disease at the time of consultation. They would not decide based on their own religious and cultural beliefs, nor length of time of consultations. Majority of the participants also said they would not be influenced by the quality of their previous experiences with medical students. These findings make it easy to deploy medical students to interact with patients without the need to first sort patients on the basis of certain characteristics. Regarding sex of the students, it is important for medical educators and attending physicians to explain to the patients beforehand the need for both male and female medical students gaining the required skills. Our findings on sex of students, previous experiences with medical students and consultation time is similar to results from other studies. For example, York et al. found no significant differences between attitudes of patients who had previous experiences with medical students and those who had not [10].

Participants from the oldest university teaching hospitals located in the capital city were less likely to be comfortable with medical students when compared with participants from teaching hospitals of the other two universities which are younger and located away from the capital city. It is not clear why acceptance of students were relatively lower in Makerere University teaching hospitals. Findings from other studies show that previous positive students-patients interactions and experience tend to increase acceptance of medical students in future consultations [10]. On the other hand, negative previous experiences tend to reduce chance of the patients accepting participation of medical students in their care [33]. Although Makerere University medical school started way back in 1923, there is limited data on perceptions of patients on the manner in which students interact with them. The attitude of patients at Makerere University teaching hospitals could relate more to the location in the capital city center and the cosmopolitan nature of the population than an intrinsic factor within the university teaching hospitals themselves. Future studies need to categorize the patients based on their frequency of previous contacts with medical students in order to delineate the influence of the quality of previous interactions on current perception of comfort with medical students’ involvement in patients’ care. In the US, patients who had had fewer contacts with medical students during their care were more likely to decline medical students’ participation in their care [20]. In addition, qualitative studies exploring perceived quality of care in previous student involvement in care may elucidate the way in which previous students’ involvement influence future acceptance and comfort with medical students.

Our participants preferred to attend care where medical students are involved; they would not want the medical students to be trained in separate university teaching hospitals different from the tertiary public hospitals where patients seek care. Our finding is similar to the study by Passaperuma et al. where they showed that majority of patients preferred teaching hospitals to nonteaching hospitals [23]. Medical students’ training could continue in the large public hospitals. If some universities start separate designated university teaching hospitals, they should keep their gates open to patients who may want to attend care where medical students are trained. The fees in such university teaching hospitals should be subsidized to avoid discriminations against the financially less privileged patients who may want to attend care in university teaching hospitals.

### Limitations

This study has some limitations inherent in the design. This was a cross sectional study; we can only appreciate associations between the socio-demographic and health systems’ factors with patients’ acceptance without asserting causality. Second, our results could be influenced by social desirability bias because data collection was conducted in the hospital setting and patients could have responded in a manner that would be socially desirable. We minimized this bias by not involving doctors and or medical students in the data collection process. Data were collected by masters of public health students and graduate research assistants who explained to the patients their status and encouraged them to provide appropriate responses without fear of any retributions. Thirdly, the tool used for data collection did not undergo psychometric testing to assess its validity and reliability. We also did not conduct exploratory factor analysis to provide insights into underlying psychological explanations for observed associations. However, we believe that the tool measured what we set out to evaluated because we adapted questions from previously validated questionnaires and piloted the tool in our local environment. We then fine-tuned the questions based on the findings from the pilot study, ensuring that the questions seek what they were designed to measure.

## Conclusions

Patients are generally comfortable with medical students’ involvement in their care; and indeed prefer to seek care in university teaching hospitals where medical students contribute to their care. In addition, clinical teachers in university teaching hospitals need to provide patients with some protected time in the absence of medical students so that matters that could not be addressed in the presence of medical students do come out. There is also need for disseminating clear messages to the public regarding the presence and roles of medical students in the university teaching hospitals so that people who come their know in advance and make deliberate choices to come there and knowingly accept or decline participation of medical students in their care. Patients need to be encouraged to provide feedback regarding the involvement of medical students in their care. These feedback can inform decisions of medical educators and preceptors on how to best train medical students without infringing on patients’ autonomy.

## Supplementary Information

**Additional file 1.** Study tool.

## Data Availability

The datasets used and/or analyzed during the current study are available from the corresponding author on reasonable request.
