# Supplementary material for: Patients’ attitudes towards involvement of medical students in their care at university teaching hospitals of three public universities in Uganda: a cross sectional study
Source: BMC Med Educ. 2022 Jul 2;22:519. doi: 10.1186/s12909-022-03576-4 (PMC9250725; doi:10.1186/s12909-022-03576-4)
Supplement: Supplementary file 1 — Additional file 1. Study tool. [file 12909_2022_3576_MOESM1_ESM.docx]

**PERCEPTIONS OF PATIENTS AND MEDICAL STUDENTS IN PUBLIC UNIVERSITY TEACHING HOSPITALS IN UGANDA REGARDING INVOLVEMENT OF MEDICAL STUDENTS IN PATIENTS’ HEALTHCARE**

**Participant Identification (ID) number: ………….**

**Interviewer Code: …………**

a) University Teaching hospital (Tick the one appropriate option)


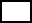
 Kawempe hospital - Makerere University


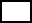
 Kiruddu hospital – Makerere University


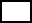
 Mbarara Regional Referral hospital – Mbarara University of Science and Technology (MUST)


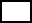
 Gulu Regional Referral hospital – Gulu University

b) Department/ward (Tick the one appropriate option)


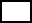
 Diabetic clinic


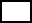
 Diabetic ward


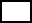
 Hypertension clinic


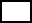
 Hypertension ward


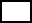
 Gynecological clinic


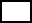
 Gynecological ward

Date of interview: Day……... Month ………… Year….......

**SECTION 1: SOCIO DEMOGRAPHIC QUESTIONS**

**Read:** Thank you for agreeing to talk with me. To start I am going to ask you some questions about yourself.

**READ ALOUD EACH QUESTION**

| **No**. | **Questions and filters** | **Response** |
| --- | --- | --- |
| 101 | How old are you  **Interviewer note:** If the participant does not know their age, ask for their date of Birth, calculate their age and write it down. If the participant doesn’t know their age or date of birth, or does not want to reveal their age tick “did not answer” | **---------** years |
|  |  | Did not answer 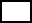 |
| 102 | What is your current relationship status?  **READ OUT ALL OPTIONS AND SELECT ONE ANSWER** | Married/ Living together with a partner 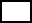 |
|  |  | Single 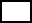 |
|  |  | Separated/Divorced 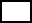 |
|  |  | Widowed 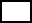 |
|  |  | 999-Refused 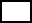 |
|  |  |  |
| 103 | What is your highest level of education?  **Interviewer note:** Use school grade and standard guide to assist you.  **TICK ONLY ONE RESPONSE** | Primary 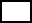 |
|  |  | Secondary 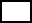 |
|  |  | Tertiary 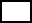 |
|  |  | University 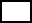 |
|  |  | No formal education 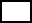 |
|  |  | 999-Refused 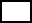 |
| 104 | What is your religion?  **TICK ONLY ONE RESPONSE** | Catholic 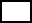 |
|  |  | Anglican 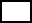 |
|  |  | Islam 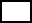 |
|  |  | Pentecostal/borne again 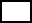 |
|  |  | Traditional believer 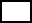 |
|  |  | Other (specify)………… 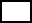 |
|  |  | 999-Refused 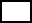 |
| 105 | a) Do you have a job/business for which you get paid or from which you earn money?  **TICK ONLY ONE RESPONSE** | Yes 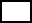 |
|  |  | No 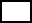 |
|  |  | 999- Refused 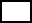 |
|  | b) If yes, what job do you do?  **PLEASE WRITE THE TITLE OR NATURE OF THE JOB** | …………………………. |
| 106 | What is your sex?  **PLEASE ASK THIS OUT ONLY WHEN YOU CANNOT DISCERN THE SEX OF THE PATIENT FROM APPEARANCE INCLUDING DRESSING** |  |
|  |  | Female 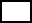 |
|  |  | Male 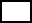 |
|  |  | Other (Please specify) …………………………… 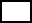 |
|  |  | 999-Refused 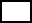 |
| 107 | a) Have you ever travelled to outside Uganda?  b) If yes, specify (name) up to three countries; | Yes 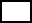 |
|  |  | No 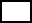 |
|  |  | 999-Refused 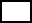 |
|  |  | …………………………… 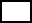 |
|  |  | ………………………… 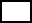 ………………………… 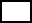 |
| 108 | When doctors come to see you for the problems that have brought you to the hospital, would you be able to tell whether some of the doctors are medical students?  **TICK ONLY ONE RESPONSE PER QUESTION** | Yes 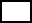 |
|  |  | No 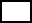 |
|  |  | Do not know 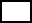 999-Refused 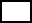 |
| 109 | If yes,  How would you tell that a doctor is a medical student?  The medical students are:  **DO NOT READ OUT THE RESPONSES BUT TICK ALL THAT THE PATIENTS SAYS**  **(KEEP PROBING - HOW ELSE MAY YOU TELL A DOCTOR IS A MEDICAL STUDENT)** | Younger 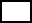 Shy 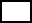 Not clear in their messages 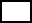 Not kind to patients 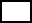 Not able to explain patients problems 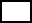 Not often exact/confident on what needs to be done 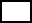 Often rough in their approaches to patients 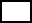 Not careful to protect patients’ privacy e.g. talk anyhow about patients’ conditions 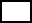 Often active, available and ready to help patients 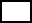 Others (please specify) ……………………………… 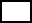 ………………………………  …………………………….  ……………………………. |
| 110 | Have you ever had a medical student present when you have been to see your doctor? | Yes 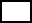 |
|  |  | No 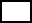 |
|  |  | Do not know 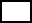 |
| 111 | If yes,  a) How many times have you had a medical student present when you have been seen by your doctor? | 1 to 2 times 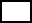 |
|  |  | 3 to 4 times 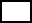 |
|  |  | 5 or more times 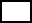 I don’t remember 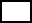 |
|  | b) When would you allow a medical student to participate in your care? | 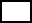 if asked by a nurse  if asked by a physician 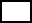 if asked by clinical medical assistants 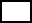 |
|  |  | if asked by a medical student 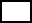 |
|  |  | 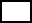 I would never allow a medical student |
|  | c) What level of participation would you allow? | Medical history only 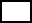 |
|  |  | History and physical exam 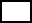 |
|  |  | 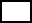 History and physical exam and procedures |
|  |  | 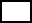 Observe physician only |
|  |  | 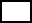 I would never allow a medical student |
| 112 | How do you feel about medical students being present while you are talking to the doctor about your problem? | Extremely uneasy 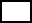 |
|  |  | Uneasy 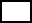 |
|  |  | 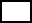 Don’t mind |
|  |  | 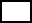 Eager |
|  |  | 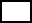 Very eager |
| 113 | Would you allow medical student(s) to be present while you are talking to the doctor about your problem? | Yes 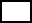 |
|  |  | Yes, if male student 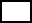 |
|  |  | 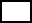 Yes, if female student |
|  |  | 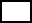 No |
|  |  | 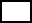 Don’t mind |
| 114 | How many medical students would you be comfortable with? | None 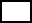 |
|  |  | 1 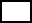 |
|  |  | 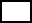 2 |
|  |  | 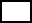 3 - 5 |
|  |  | 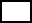 Even more than 5 |
|  |  | 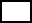 Don’t mind |
| 115 | Would you allow a medical student to observe or watch while the doctor examines you? | Yes in all cases 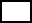 |
|  |  | Yes, depending on which part of my body is being examined 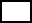 |
|  |  | 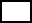 Yes, if male student |
|  |  | 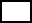 Yes, if male student, and depending on which part of my body is being examined |
|  |  | 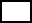 Yes, if female student |
|  |  | 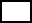 Yes, if female student, and depending on which part of my body is being examined |
|  |  | No in all cases 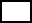 |
| 116 | Would you allow a medical student to examine you with the doctor supervising them? | Yes in all cases 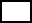 |
|  |  | Yes, depending on which part of my body is being examined 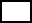 |
|  |  | 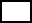 Yes, if male student |
|  |  | 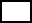 Yes, if male student, and depending on which part of my body is being examined |
|  |  | Yes, if female student |
|  |  | Yes, if female student, and depending on which part of my body is being examined |
|  |  | No in all cases |
| 117 | Would you permit medical students to examine you without the presence of a doctor? | Yes in all cases |
|  |  | Yes, depending on which part of my body is being examined |
|  |  | Yes, if male student |
|  |  | Yes, if male student, and depending on which part of my body is being examined |
|  |  | Yes, if female student |
|  |  | Yes, if female student, and depending on which part of my body is being examined |
|  |  | No in all cases |
| 118 | From the list below, please tick the 2 main reasons for your decision about whether medical students should be present at the consultation or not? | My cultural beliefs |
|  |  | My religious beliefs |
|  |  | It will take more time for the consultation |
|  |  | My personality |
|  |  | My condition is not too bad |
|  |  | Prior experience with medical students |
|  |  | Gender of medical student |
|  |  | Quality of care may be affected |
|  |  | Other (please specify)…………………….. |
| 119 | How important for the training of future doctors do you think it is that medical students are present while patients are talking to the doctor about their problem? | Very important |
|  |  | Important |
|  |  | Not sure |
|  |  | Not so important |
|  |  | Unnecessary |
| 120 | How important for the future training of doctors do you think it is that medical students examine patients? | Very important |
|  |  | Important |
|  |  | Not sure |
|  |  | Not so important |
|  |  | Unnecessary |
| 121 | Would you permit medical students to take your medical history and personal details from you without the presence of a doctor? | Yes only males |
|  |  | Yes only females |
|  |  | Yes both males and females |
|  |  | Neither males nor females |
| 122 | Would you permit medical students to read your medical file? | Yes |
|  |  | No |
|  |  | Don’t know |
| 123 | Would you permit medical students to be present in the outpatient clinic if you were having a consultation with your doctor? | Yes only males |
|  |  | Yes only females |
|  |  | Yes both males and females |
|  |  | Neither males nor females |
| 124 | Would you permit medical students to be present in the ward rounds if you were admitted in the same ward? | Yes only males |
|  |  | Yes only females |
|  |  | Yes both males and females |
|  |  | Neither males nor females |
| 125 | Would you permit medical students to be present in the operation room if you were having a surgery? | Yes only males |
|  |  | Yes only females |
|  |  | Yes both males and females |
|  |  | Neither males nor females |
| 126 | Would you permit medical students to be present while you’re having diagnostic/other procedures (e.g. drawing blood, inserting catheter, endoscopy .etc)? | Yes only males |
|  |  | Yes only females |
|  |  | Yes both males and females |
|  |  | Neither males nor females |
| 127 | Would you permit medical students to perform diagnostic/other procedures on you (e.g. drawing blood, inserting catheter, endoscopy .etc)? | Yes only males |
|  |  | Yes only females |
|  |  | Yes both males and females |
|  |  | Neither males nor females |

|  | Regarding teaching hospitals and care for patients | |
| --- | --- | --- |
| 128 | Rank the following situations based on what your preference would be from **the most preferred (1^st^) to the least preferred (5^th^)** | |
| a) | Having teaching hospitals separate from public hospital  1^st^ (**most preferred**) 2^nd^ 3^rd^ 4^th^ 5^th^ (**least preferred**) | |
| b) | Having medical students in only national referral hospital  1^st^ (**most preferred**) 2^nd^ 3^rd^ 4^th^ 5^th^ (**least preferred**) | |
| c) | Having medical students in only regional referral hospital  1^st^ (**most preferred**) 2^nd^ 3^rd^ 4^th^ 5^th^ (**least preferred**) | |
| d) | Having medical students in both national and regional referral hospital  1^st^ (**most preferred**) 2^nd^ 3^rd^ 4^th^ 5^th^ (**least preferred**) | |
|  |  |  |

129. Please score according to importance. Each of the following factors which you may consider when making decisions whether medical students should participate in your care during consultation and examination. Tick only one most appropriate option.

1. Not important

2. Slightly Important

3. Important

4. Moderately important

6. Very important

| Considerations | 1 | 2 | 3 | 4 | 5 | 6 |
| --- | --- | --- | --- | --- | --- | --- |
| **Students attributes** | | | | | | |
| Student is respectful |  |  |  |  |  |  |
| Student is polite |  |  |  |  |  |  |
| Student is caring |  |  |  |  |  |  |
| Student is gentle |  |  |  |  |  |  |
| Student is neat and clean |  |  |  |  |  |  |
| Student listens to me |  |  |  |  |  |  |
| It is easy to talk to the student |  |  |  |  |  |  |
| **Patients’ needs** | | | | | | |
| Need to t5alk to my privately with my doctor |  |  |  |  |  |  |
| I only see my doctor once a year |  |  |  |  |  |  |
| **Students’ gender** | | | | | | |
| Student is female |  |  |  |  |  |  |
| Student is male |  |  |  |  |  |  |

**Thank you very much for participating in this survey**
